# Identification and evaluation of reference genes in the Chinese white wax scale insect *Ericerus pela*

**DOI:** 10.1186/s40064-016-2548-z

**Published:** 2016-06-21

**Authors:** Shu-Hui Yu, Pu Yang, Tao Sun, Qian Qi, Xue-Qing Wang, Dong-Li Xu, Xiao-Ming Chen

**Affiliations:** Research Institute of Resources Insects, Chinese Academy of Forestry, Key Laboratory of Cultivating and Utilization of Resources Insects of State Forestry Administration, Kunming, 650224 China

**Keywords:** Chinese white wax scale insect, Developmental stages, Reference gene, Temperature treatments, Tissues

## Abstract

**Background:**

The Chinese white wax scale insect, *Ericerus pela*, is a well-known resource insect. The females and males are dramatically distinct at each developmental stage. We sought to identify suitable reference genes to use as internal controls in molecular research on *E. plea*.

**Results:**

geNorm, RefFinder and Normfinder analyses showed that ßTub-2 was the best reference gene throughout different developmental stages; SdhA-1 was the most stable reference gene in different tissues, and ßTub-1 was the most reliable reference gene under treatment with different temperatures. The results also showed that the optimal number of reference genes for analyzing target gene expression levels in the three experimental conditions was two.

**Conclusions:**

The identified reference genes are suitable reference genes for normalization in RT-qPCR of *E. pela* samples.

## Background

The Chinese white wax scale insect, *Ericerus pela*, is a well-known resource insect species, owing to its role in wax production and has been bred in China for over one thousand years. White wax, produced by the male second-instar larvae, is widely used in the pharmaceutical, chemical, and cosmetics industries. However, female *E. pela* cannot produce wax. In addition, females and males are dramatically distinct at each developmental stage. The males undergo holometabolous-like development, developing through egg, first- and second-stage nymph, pupal (prepupal and pupal), and adult stages and emerging as winged adults. The second-instar male nymphal period lasts 2 months, which is a relatively long period, whereas the male adult period lasts only 1 week because the males die immediately after mating (Chen 2011). In contrast, the females undergo neotenous development, developing through egg, first- and second-stage nymph, and adult stages. The female adult period is approximately 8 months and is the longest developmental stage in *E. plea*. *E. pela* is widely distributed from the subtropics to temperate areas in China, Korea, and Japan. Individuals can survive a high temperature of 44.0 °C and a low temperature of −30.4 °C in these areas (Chen 2011).

Many ecological and biological studies have been performed to determine the biological characteristics and wax production of *E. pela* in recent decades, because of the species’ economic importance. Recently, extensive research has delved deeper into this topic, and many molecular biology-focused studies have been performed using *E. pela*, such as the selection of functional genes and studies on the molecular mechanisms underlying the physiological functions of *E. pela* (Yang et al. 2012; Liu et al. 2014). However, a comprehensive study on *E. pela* reference genes is lacking. To determine suitable reference genes for fluorescence real-time quantitative polymerase chain reaction (RT-qPCR) experiments in *E. pela*, we selected fifteen commonly used reference genes (Table 1) and tested their expression levels using RT-qPCR at different developmental stages, in different tissues, and under different temperature stresses. Expression stability was analyzed using three software applications: geNorm (Vandesompele et al. 2002), Normfinder (Andersen et al. 2004), and RefFinder (Xie et al. 2012; Fu et al. 2013). These software applications have been widely used to select reference genes (Zhu et al. 2012; Shivhare and Lata 2016). The objective of this study was to identify suitable reference genes to use as internal controls in molecular research on *E. pela*.

**Table 1:** The relevant parameters of the fifteen reference genes

Gene symbol	Gene name	Primer sequences (5′–3′)	Amplicon length (bp)	Amplicon Tm (°C)	Regressionco efficient (R^2^)	Amplification efficiency (℅)
ACT-1	Actin	F: ATTGTAGGTCGTCTCGTGG R: AGTACTCCGTATGGATCG	158	82	0.982	102.0
ACT-2	Actin	F: GAATCGCTCTCCTCCGACTTTGAT R: GATCTACCATGTACCCAGGATTAC	142	84.5	0.999	102.8
Tub	Tubulin	F: CTTCATGGGGTAGTAAACGCACTA R: GACAAATCGCGTGATGCAGCTGCAT	102	80.0	0.994	91.6
βTub-1	β-Tubulin	F: CTCATCCATACCCTCACCGGTGT R: ACCAAGAAGCTACTGCCGACG	155	81.5	0.992	102.6
βTub-2	β-Tubulin	F: AGTTCGGCACCTTCTGTGTAATGC R: GTTCCATCCCCAAAGGTCTCTGAT	391	78.5	0.998	94.0
SdhA-1	Succinate dehydrogenase, subunit A	F: CTAATGTTTCTACCAAGTCGGTAT R: GAATCGCGCGGTGCTCATGCTCGA	107	76	0.997	95.5
SdhA-2	Succinate dehydrogenase, subunit A	F: CTAATGCGGCATTAATACCACCT R: CACTGTTAAAGGATCCGATTGGT	86	75	0.997	94.8
SdhA-3	Succinate dehydrogenase, subunit A	F: GTATGCCACCCATATTGTAATGTAC R: CATGGAGCCAATCGATTGGGTGCCA	148	79	0.997	95.6
RP II	RNA polymerase-II transcription	F: ATTTTCTTCGCCCTCTTGCA R: ATCGAGGCCATCTGCAAGG	114	78.5	0.997	97.2
mRpL50-1	Mitochondrial ribosomal protein L50	F: CAGTATCAGGGTGGAATCTGTGATA R: GATGCCGGTAGTCCATGGCACGAT	135	75.5	0.995	95.4
mRpL50-2	Mitochondrial ribosomal protein L50	F: AGTCCAACCATGGCCCTTATTAGCGA R: GTCATGTCAGCTTCTTCGGATGAGTG	148	78.5	0.999	91.0
mRpL15	Mitochondrial ribosomal protein L15	F: GTACGCAGTATTACGCTTGGGCATG R: CATGTTTCATTGCGTTGACGATTCT	135	77.5	0.998	92.7
UBQ-1	Ployubiquitin	F: CTGAACGTGAATTTACCTCCTCTGTA R: GAAGCTGCTGAGGTCCTTCAATCAA	256	78.5	0.998	92.2
UBQ-2	Ployubiquitin	F: CATACGTGCTAGTCCAATGCTCAGC R: GATGCGATATGGATCAAGCACTGGT	110	78	0.975	94.8
Myo	Myosin	F: ACGATCTCCCATCGAGATCAACA R: GATTACACCGAATTCACTCGCAT	106	76.5	0.991	99.0

## Methods

### Sample preparation

*Ericerus pela* individuals at different developmental stages (15 stages) were collected and placed in 1.5-ml centrifuge tubes (50–100 mg each tube), including first-instar female nymphs, second-instar female nymphs, female adults, first-instar male nymphs, second-instar male nymphs, prepupae, pupae and male adults, as well as eggs. The second-instar male nymphal stage and female adult stage extend for a long period of time, and individuals in these stages vary in body size and physiological activity. Thus, each of these samples was divided into four different time points. The eggs were a mixture of females and males because it was difficult to distinguish female eggs from male eggs.

The various tissues examined (11 tissues) included the cuticle, fat body, alimentary canal, and Malpighian tubules in females and males; the ovary and abdomen in females; and the testis in males.

The different temperature treatments (9 treatments) included female adults and second-instar male nymphs exposed to temperatures of −20, −10, 0, 10, 25, 30, 35, 40, and 45 °C.

### RNA extraction and RT-qPCR

Total RNA was extracted from each pooled sample using TRIzol (Invitrogen, Carlsbad, California U.S.) protocol. The resultant RNA concentrations and quality were determined using an Agilent 2100 Bioanalyzer (Agilent Technologies, California, U.S.). First-strand cDNA synthesis was performed in accordance with the manual *First*-*Strand cDNA Synthesis Using M*-*MLV* (Invitrogen, Shanghai, China).

### Primer design

Fifteen commonly used reference genes were selected as candidate reference genes, and the SRA accession number of the transcriptome that contained the fifteen nucleotide sequences was SRA047286.1. Using the primer design software Primer 5.0, we designed RT-qPCR primers for each of the 15 reference genes (Table 1). The design principles included the following: product length between 80 bp and 300 bp; primer length between 18 bp and 25 bp; GC content between 40 and 60 %; bases evenly distributed; and no more than three successive complementary bases between primers and primer pairs. Additionally, the melting temperature (Tm) should be between 58 and 62 °C, the products should be single products, and primer dimers should be avoided. The primer’s overall Gibbs Free Energy (∆G) value from the 5′-end should be higher than that from the 3′-end. Finally, the PCR products of the primers were synthesized by Kunming Shuoqing Biological Technologies Ltd.

### RT-qPCR

The RT-qPCR reaction was performed using 2× Supermix (SsoFast™EvaGreen^®^. Bio-Rad, Hercules, California, U.S.). The PCR procedures included an initial step at 95 °C for 3 min, followed by 39 reaction cycles at 95 °C for 10 s and 55 °C for 20 s. Next, the plate was read, and melting curves were drawn (65–95 °C, +0.5 °C/5 s) in the PCR instrument (BIO-RADCFX96™ Real-Time System. Bio-Rad, Hercules, California, U.S.). Three replicate experiments were performed. The negative (i.e., without adding the template) and blank (i.e., without adding the template and primer) controls were generated simultaneously.

### Data processing

The amplification efficiency and specificity for each gene were analyzed. The threshold cycle (Ct) values obtained for each of the reference genes from different samples were statistically evaluated using the software applications geNorm, Normfinder, and RefFinder (Vandesompele et al. 2002; Andersen et al. 2004; Xie et al. 2012; Fu et al. 2013).

### Ethics and consent

No specific permits were required for the described field studies. The white wax scale insect was bred at the Research Institute of Resource Insects. One individual can produce thousands of offspring. No specific permissions were required for these locations/activities.

## Results

### Primer amplification efficiency and specificity

**Fig. 1:**
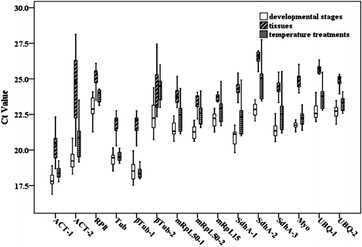
Distribution of the cycle threshold (Ct) values of the fifteen reference genes in the fifteen developmental stages, eleven tissues, and nine temperature treatments. The x-axis shows the fifteen reference genes, and the y-axis shows the Ct values

The amplification efficiency ranged from 91.0 to 102.8 %, and the correlation coefficient (R^2^) was above 0.975. Each melting curve showed a single peak, and no fluorescent signals were observed in the negative control, which indicated good specificity. The average Ct values of the fifteen reference genes were all less than 30, and all of the reference genes were therefore analyzed and evaluated (Fig. 1).

### Different developmental stages

**Fig. 2:**
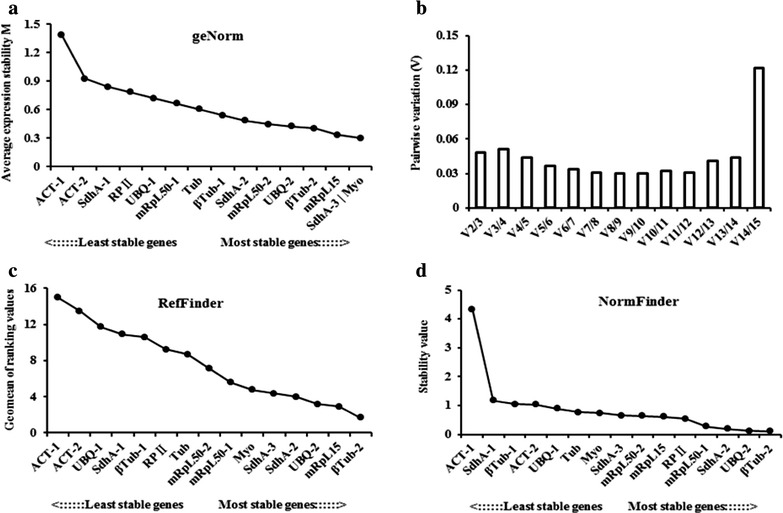
Stability ranking of the reference genes during the developmental stage analysis performed using geNorm, RefFinder, and Normfinder software. **a** Stability ranking of the reference genes analyzed with geNorm; **b** V values for pair-wise variation between two normalization factors generated by geNorm; **c** stability ranking of the reference genes analyzed with RefFinder; **d** stability ranking of the reference genes analyzed with Normfinder

geNorm analysis indicated that the M values for all of the reference genes were below 1.5. There were seven genes with M values below 0.5, seven genes with M values between 0.5 and 1.0, and only one gene with an M value between 1.0 and 1.5. The ranking of the reference genes is shown in Fig. 2a. The most stable genes were SdhA-3 and Myo. The stability values for ßTub-2 and mRpL15 were only slightly higher than those of SdhA-3 and Myo (Fig. 2a).

The V values for the pair-wise variation between two normalization factors generated by geNorm were below the 0.15 threshold. The V2/3 value was less than 0.15, which indicated that a third reference gene did not exert a significant normalizing effect on this experiment. Therefore, the recommended number of reference genes for this experimental situation was two (SdhA-3 and Myo; Fig. 2b).

ßTub-2 was the most stable gene in the RefFinder and NormFinder results (Fig. 2c, d). ßTub-2 was also indicated as a stable gene with an M value very similar to those of the two most stable genes (SdhA-3 and Myo) in the geNorm results (Fig. 2a), thus suggesting that ßTub-2 was the best independent gene in different developmental stages.

### Different tissues

**Fig. 3:**
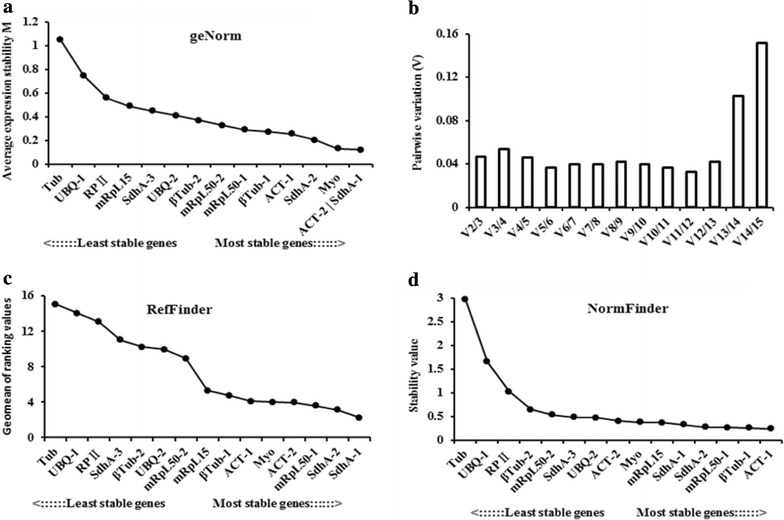
Stability ranking of the reference genes in the analysis of different tissues with geNorm, RefFinder, and Normfinder software. **a** Stability ranking of the reference genes analyzed with geNorm; **b** V values for pair-wise variation between two normalization factors generated by geNorm; **c** stability ranking of the reference genes analyzed with RefFinder; **d** stability ranking of the reference genes analyzed with Normfinder

According to the results of the geNorm analysis, the M values of all the genes were below 1.5. There were 12 genes with an M value below 0.5, two genes with M values between 0.5 and 1.0, and only one gene with an M value between 1.0 and 1.5. The recommended genes are shown in Fig. 3a. SdhA-1 and ACT-2 were the most stable genes.

The V values were less than 0.15, except for V14/15. Because the V2/3 value was less than 0.15, the optimal number of reference genes was two (i.e., SdhA-1 and ACT-2; Fig. 3b).

RefFinder recommended the same five suitable genes (i.e., SdhA-1, SdhA-2, mRpL50-1, ACT-2, and Myo) indicated by the geNorm analysis results but in a different ranking order, except for mRpL50-1 (Fig. 3c).

Three of these genes (SdhA-1, SdhA-2, and mRpL50-1) were also recommended by the NormFinder analysis results with approximate stability values (Fig. 3d).

The integrated results indicated that SdhA-1 was the best independent reference gene in different tissues.

### Different temperature treatments

**Fig. 4:**
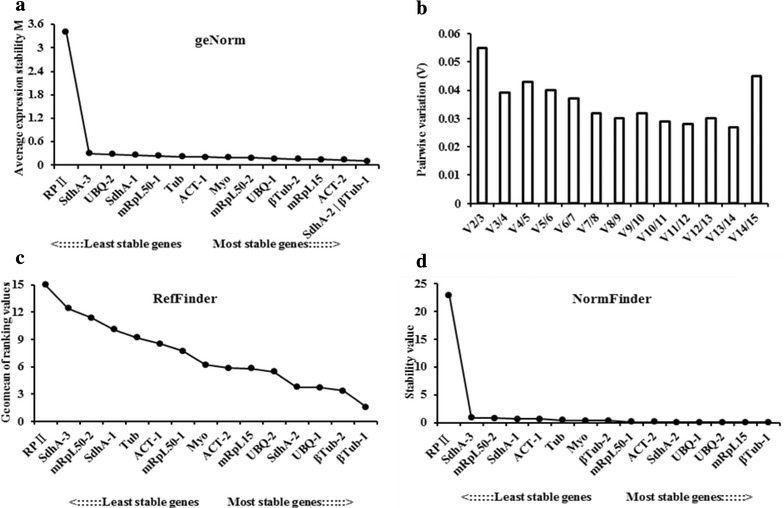
Stability ranking of the reference genes in the analysis of different temperature treatments with geNorm, RefFinder, and Normfinder software. **a** Stability ranking of the reference genes analyzed with geNorm; **b** V values for pair-wise variation between two normalization factors generated by geNorm; **c** stability ranking of the reference genes analyzed with RefFinder; **d** stability ranking of the reference genes analyzed with Normfinder

geNorm analysis demonstrated that the RPII gene exhibited the highest M value, which was far greater than 1.5, whereas the M values of the other 14 genes were below 0.5, which demonstrated that they were suitable reference genes in the samples subjected to different temperature treatments (Fig. 4a). The rank of the recommended genes is shown in Fig. 4a. ßTub-1 and SdhA-2 were the two most stable genes (Fig. 4a).

All of the V values were below 0.15. The V2/3 value was less than 0.15, and the recommended number of reference genes was therefore two (ßTub-1 and SdhA-2; Fig. 4b).

ßTub-1 was also suggested to be the most stable gene by RefFinder and NormFinder (Fig. 4c, d) and by the geNorm analysis (Fig. 4a). PRII was the least stable gene in all three software analyses.

We concluded that ßTub-1 could be considered the most stable reference gene under treatments involving different temperatures. However, PRII is not recommended.

## Discussion

RT-qPCR is the most commonly used method for measuring gene expression levels. RT-qPCR quantification requires the use of reference genes to normalize the data under different experimental conditions. However, it was impossible to find one gene suitable for all experimental conditions. Reference gene selection studies have been performed on many insects at different developmental stages, in different tissues, using different treatments and different populations. These studies have provided good reference genes for normalizing experimental procedures in RT-qPCR experiments. Regarding the selection of reference genes in scale insects, no relevant study has been reported to date.

In the present study, we evaluated 15 *E. pela* housekeeping genes by using RT-qPCR technology. ACT and Tub are cytoskeleton proteins (Zarrouk et al. 2015), and ACT and Myo form the filaments of the muscle (Li et al. 2016). SdhA is a component of electron transport chain complexes (Cao et al. 2016). RP II catalyzes DNA transcription and synthesis of mRNA precursors (Sims et al. 2004). mRpL aids in mitochondrial protein biosynthesis (Goldschmidt-Reisin et al. 1998). UBQ plays a key role in the ubiquitin–proteasome system. We analyzed and evaluated the expression stability of the 15 reference genes during different developmental stages, in different tissues, and under different temperature treatments by using different software programs: geNorm, Normfinder, and RefFinder. The geNorm analysis showed that the M values were all below 1.5, except for RPII in the different temperature treatments. A value of 1.5 is considered to be the threshold for a reliable reference gene in geNorm analysis (Storch et al. 2015). According to Hellemans et al. (2007) the M value threshold is 0.5 for homogeneous samples and 1.0 for heterogeneous samples. There were at least more than seven reference genes that were identified as reliable reference genes in each of the three experiments.

The three applied software programs use different statistical methods to evaluate and analyze reference genes. Although these programs produced different rankings for the reference genes in different developmental stages and different tissues, the stable values of these reference genes were very similar. The integrated results provided similar relatively stable reference genes. We concluded that ßTub-2 and SdhA-1 were the best independent genes in different developmental stages and different tissues separately. Notably, in the different temperature treatments, the three programs all recommended ßTub-1 as the most stable reference gene and PRII as the least stable reference gene.

Similarly, Tub has previously been suggested to be the most suitable reference gene at different developmental stages in the white-backed planthopper, *Sogatella furcifera* (An et al., 2015). However, Tub shows variations in different developmental stages in *Tribolium castaneum* (Sang et al., 2015).

SdhA has been identified as the least stable gene in the Australian plague locust, *Chortoicetes terminifera* (Chapuis et al., 2011). The suitable reference genes for different tissues in various insects generally vary (An et al. 2015; Pan et al. 2015; Wang et al. 2015).

However, RP II has previously been found to be the most stable gene under starvation stress or UV irradiation stress conditions in *Toxoptera citricida* (Hemiptera, Aphidiae) (Shang et al., 2015). These results suggest that reference gene studies should be performed for each experimental system rather than adopting reference genes from other species.

Moreover, geNorm indicated that the optimal number of reference genes in the three experimental conditions was two. Using multiple reference genes with the lowest geNorm values may provide more a conservative estimation of gene expression levels.

## Conclusion

The integrated analyses from the three software programs (geNorm, Normfinder, and RefFinder) recommended ßTub-2 as the best reference gene during different developmental stages, SdhA-1 as the best reference gene in different tissues, and ßTub-1 as the best reference gene under treatments involving different temperatures. The recommended number of reference genes in the three experimental conditions was two. These results provided reliable reference genes that are suitable normalizers for further RT-qPCR experiments in *E. pela*.
